# High Expression of ROMO1 Aggravates the Malignancy of Hepatoblastoma

**DOI:** 10.1155/2021/2341719

**Published:** 2021-09-01

**Authors:** Jiangfeng Lv, Yan Wu, Wei Li, Huaping Fan

**Affiliations:** ^1^Department of Clinical Laboratory, Jinan City People's Hospital, Jinan People's Hospital Affiliated to Shandong First Medical University, Jinan, Shandong 271199, China; ^2^Department of Clinical Laboratory, Yantai Yuhuagding Hospital Affiliated to Qingdao University, Yantai, Shandong 264000, China; ^3^Department of Oncology (II), Qingdao Central Hospital Affiliated to Qingdao University, Qingdao, Shandong 266042, China; ^4^Department of Pediatrics, Yantai Maternal and Child Health Care Hospital, Yantaishan Hospital, Yantai, Shandong 264000, China

## Abstract

Hepatoblastoma (HB) is a kind of tumor that occurs frequently in children and is highly malignant. Here, the function of ROS modulator 1 (ROMO1) was identified in the development of HB. In this study, the mRNA expression of ROMO1 was measured by RT-qPCR. Colony formation assay, MTT assay, and flow cytometric analysis were applied to detect cell viability. The cell migrative and invasive ability was measured by wound healing and transwell assays. Tumor xenografts were performed to examine tumor growth. The results showed that upregulation of ROMO1 was identified in liver hepatocellular carcinoma (LIHC) tissues and predicted poor prognosis in LIHC patients. And ROMO1 expression was also increased in HB tissues and cells. Functionally, ROMO1 knockdown restrained cell viability, migration, and invasion in HB. In addition, knockdown of ROMO1 was found to suppress tumor formation *in vivo*. In conclusion, upregulation of ROMO1 promotes tumor growth and cell aggressiveness in HB.

## 1. Introduction

Hepatoblastoma (HB) develops from hepatocyte precursor cells and is a malignant embryonic tumor with multiple differentiation modes [1]. The cause of HB is not clear. Some genetic diseases and congenital factors may be related to the occurrence of HB [2]. HB is the most common liver malignant tumor in children, accounting for 90% of the primary malignant tumors of the liver in children. The incidence of HB is about 0.7 to 1/100 million, and the male-to-female ratio is approximately (1.5 to 2) : 1 [3]. Infants and young children, especially those who have been infected with hepatitis B virus, have an increased risk of HB [4]. Additionally, the early symptoms of HB are not obvious, and the child usually looks good. After that, the tumor grows rapidly, and an abdominal mass may appear, which is the most common symptom of HB [5]. The treatment of HB is mainly surgery combined with chemotherapy. If the patient is actively treated, the overall survival rate of HB is about 80%, and the prognosis is good [6]. Therefore, early detection, early diagnosis, and early treatment are extremely important for improving the survival rate of patients with HB.

In the past few years, many RNAs and genes have been found to be involved in the development of HB. For example, microRNA-26-5p functioned as a new inhibitor of HB by repressing the LIN28B-RAN-AURKA pathway [7]. LncRNA CRNDE promoted the angiogenesis of HB by regulating the miR-203/VEGFA axis [8]. Circ-STAT3 promoted HB progression through acting as a sponge for miR-29a/b/c-3p [9]. In addition, DPEP1 has been reported to promote HB progression by the PI3K/Akt/mTOR pathway [10]. In this study, the role of ROMO1 in HB was investigated.

ROS modulator 1 (ROMO1) is well known to regulate intracellular ROS production. And in many human cancers, the important role of ROMO1 has been found. For example, ROMO1 predicted unfavorable prognosis in colorectal cancer patients [11]. And ROMO1 was also found to regulate ROS production and cellular growth in gliomas [12]. In addition, it has been reported that ROMO1 overexpression predicted worse survival in patients with NSCLC [13]. More importantly, overexpression of ROMO1 was found to promote the production of ROS and hepatic tumor cell invasion [14]. However, the function of ROMO1 in HB has not been reported in previous studies.

To investigate whether ROMO1 is involved in the pathogenesis of HB, the expression of ROMO1 in HB tissues and cells was examined. At the same time, the effect of ROMO1 on tumor growth and cell aggressiveness was also identified in HB. ROMO1 may be a potential biological target for HB.

## 2. Materials and Methods

### 2.1. Clinical Samples

HB tissues and adjacent normal tissues were collected from 18 patients in Yantai Maternal and Child Health Care Hospital, Yantaishan Hospital, between January 2014 and December 2020. The average age of these patients was 4.8 years between 2 and 9 (from 2 to 9 years old). Among them, there were 11 male and 7 female patients. Written informed consent was obtained from every patient or their direct relatives. Our research was approved by the Ethics Committee of Yantai Maternal and Child Health Care Hospital, Yantaishan Hospital.

### 2.2. Bioinformatics Analysis of ROMO1

The expression of ROMO1 in human cancers was analyzed in the GEPIA (http://gepia.cancer-pku.cn/) database. The expression level and prognosis of ROMO1 in liver hepatocellular carcinoma (LIHC) were analyzed by box plots and survival plots.

### 2.3. Cell Cultures

Normal hepatocyte cell lines LO2 and HepG2 and HuH-6 HB cells were purchased from the Chinese Academy of Sciences Cell Bank. LO2 and HuH-6 cells were incubated in the DMEM medium with 10% FBS at 37°C under an atmosphere with 5% CO_2_.

### 2.4. Cell Transfection

ROMO1 siRNAs (si-ROMO1) and negative control (si-NC) were purchased from RiboBio (Guangzhou, China). For *in vitro* experiments, si-ROMO1 and si-NC were transfected into HuH-6 cells using Lipofectamine 3000 (Invitrogen, CA, USA). For *in vivo* experiments, ROMO1 knockdown lentivirus (sh-ROMO1) and empty lentivirus control (Mock) were constructed into HuH-6 cells.

### 2.5. RT-qPCR

Total RNA of ROMO1 was isolated from HB tissues and cells by TRIzol reagent (Invitrogen, CA, USA). cDNA was synthesized using the High-Capacity cDNA Reverse Transcription Kit (Applied Biosystems, CA, USA). RT-qPCR assay was performed on the StepOnePlus Real-Time PCR System by using the Power SYBR Green PCR Master Mix (Applied Biosystems). GAPDH was an internal control. ROMO1 expression was analyzed using the 2^-△△Cq^ method.

### 2.6. MTT Assay

The transfected HuH-6 cells were reseeded in a 96-well plate (4 × 10^3^ cells/well). MTT solution (0.5 mg/ml) was used to incubate these cells based on the MTT assay. The absorbance was detected at 490 nm with a spectrophotometer.

### 2.7. Colony Formation Assay

The transfected HuH-6 cells were seeded into 6-well plates (4 × 10^3^ cells/well). Next, the cells were cultured in a cell incubator for 10 days. Then, the colonies were fixed with 4% paraformaldehyde and stained with 0.1% crystal violet. Finally, cell colonies were counted and photographed.

### 2.8. Flow Cytometric Analysis

Annexin V-FITC/propidium iodide Apoptosis Detection Kit I (BD Biosciences, Franklin Lakes, NJ, USA) was used to detect apoptotic cells. In brief, 0.25% EDTA-free trypsin was added to digest HuH-6 cells. Then, the cells were resuspended in 100 mL binding buffer. Next, 5 ml Annexin V-FITC and PI were added to incubate these cells for 30 min at 37°C in the dark. Finally, flow cytometry (Becton, Dickinson and Company, CA, USA) was applied to measure the apoptotic cells.

### 2.9. Wound Healing Assay

HuH-6 cells (4 × 10^5^ cells/well) were seeded into six-well plates. After reaching 90% confluence, a 1 mm-wide wound was made by a 200 mL sterile needle tip. The wound area was measured and photographed under a microscope every 24 h.

### 2.10. Transwell Assay

Transwell chamber (10 *μ*m pore membrane, BD Biosciences) was used to assess the cell migrative and invasive ability. The transfected HuH-6 cells (5 × 10^3^ cells/well) were added to the upper chamber. DMEM medium with 20% FBS was added in the bottom well. After 24 h, HuH-6 cells in the chamber were fixed with methanol for 30 min and stained with crystal violet for 15 min. Migrated cells were observed and counted under a light microscope. For cell invasion, Matrigel (BD Biosciences) was added in the top side of the upper chamber. Besides this, the whole experiment was similar to the cell migration method.

### 2.11. Tumor Xenografts

The male BALB/c nude mice (4–6 weeks) were obtained from Beijing Vital River Laboratory (Beijing, China). HuH-6 cells with sh-ROMO1 and Mock were subcutaneously injected into the lower flank of nude mice. Tumor growth was detected every 4 days. Mice were euthanized after 28 days. Finally, tumors were dissected and weighed. Before this study, the Animal Health Committee of Yantai Maternal and Child Health Care Hospital, Yantaishan Hospital, approved this experiment.

### 2.12. Statistical Analysis

GraphPad Prism 6 software was used for statistical analysis. Data were expressed as mean ± SD. Difference was analyzed by Student's *t*-test or one-way ANOVA, with the significant level specified as *P* < 0.05.

## 3. Results

### 3.1. Upregulation of ROMO1 Is Identified in LIHC Tissues and Predicts Poor Prognosis in Liver Hepatocellular Carcinoma (LIHC) Patients

**Figure 1:**
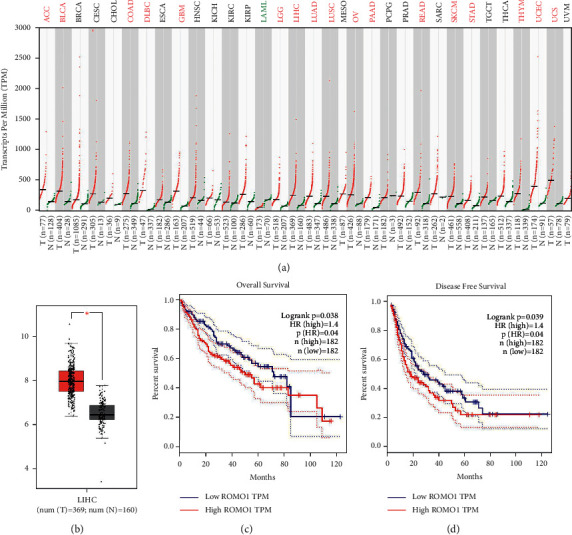
Upregulation of ROMO1 is identified in LIHC tissues and predicts poor prognosis in LIHC patients. (a) ROMO1 expression in human cancers was analyzed in the GEPIA database. (b) ROMO1 expression in LIHC tissues. (c, d) Analysis of OS and DFS rates in LIHC patients with high or low ROMO1 expression. ^*∗*^*P* < 0.05.

First, the expression of ROMO1 in different human cancers was analyzed by using the GEPIA database (TCGA and GTEX). We found that ROMO1 expression was increased in most cancers (*P* < 0.05, Figure 1(a)). At the same time, upregulation of ROMO1 was also found in LIHC tissues in comparison with normal tissues (*P* < 0.05, Figure 1(b)). In addition, LIHC patients with higher ROMO1 expression were found to have lower overall survival (OS) and disease-free survival (DFS) than patients with low ROMO1 expression (*P* < 0.05, Figures 1(c) and 1(d)). These results demonstrate that ROMO1 is upregulated in LIHC tissues. And upregulation of ROMO1 predicts poor prognosis in LIHC patients.

### 3.2. ROMO1 Expression Is Also Increased in HB Tissues and Cells

**Figure 2:**
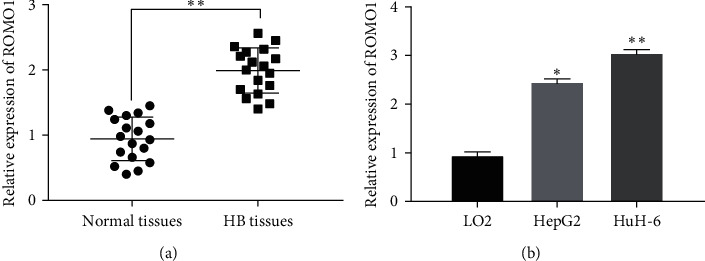
ROMO1 expression is also increased in HB tissues and cells. (a) ROMO1 expression was detected in HB tissues and normal tissues by RT-qPCR. (b) ROMO1 expression was detected in normal hepatocyte cell lines LO2 and HepG2 and HuH-6 HB cells. ^*∗*^*P* < 0.05 and ^*∗∗*^*P* < 0.01.

Although the expression of ROMO1 in LIHC is known, the alternation of ROMO1 expression in HB remains unclear. Thus, ROMO1 mRNA expression was detected in 18 HB tissues in our study. Compared with normal tissues, upregulation of ROMO1 was detected in HB tissues (*P* < 0.05, Figure 2(a)). In addition, ROMO1 expression in HB cells was also detected. Compared with normal hepatocyte cell line LO2, ROMO1 was upregulated in HuH-6 and HepG2 HB cells (*P* < 0.05, Figure 2(b)). Based on these results, we infer that ROMO1 may be involved in the pathogenesis of HB. We also found that the difference of ROMO1 expression in HuH-6 cells was more significant than HepG2 cells (Figure 2(b)). Therefore, HuH-6 was selected to explore the function of ROMO1 in HB.

### 3.3. Knockdown of ROMO1 Restrains Cell Viability in HB

**Figure 3:**
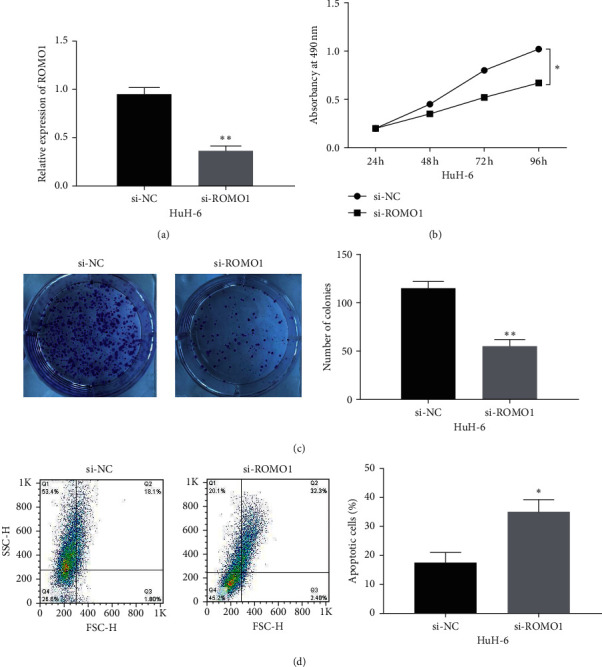
Knockdown of ROMO1 restrains cell viability in HB. (a) ROMO1 expression was detected in HuH-6 cells with si-ROMO1 or si-NC. (b) Cell proliferation was detected by the MTT assay in HuH-6 cells with si-ROMO1 or si-NC. (c) Colony-forming ability was assessed in HuH-6 cells with si-ROMO1 or si-NC. (d) Annexin V-FITC/PI flow cytometry was used to evaluate apoptosis in HuH-6 cells with si-ROMO1 or si-NC. ^*∗*^*P* < 0.05 and ^*∗∗*^*P* < 0.01.

Next, si-ROMO1 or si-NC was transfected into HuH-6 cells. RT-qPCR showed that ROMO1 expression in the si-ROMO1 group was reduced in comparison with the si-NC group (*P* < 0.01, Figure 3(a)). Functionally, ROMO1 downregulation suppressed cell proliferation in HuH-6 cells compared to the si-NC group (*P* < 0.01, Figure 3(b)). Meanwhile, we found that the colony-forming ability of HuH-6 cells was reduced after ROMO1 knockdown (*P* < 0.05, Figure 3(c)). To further explore the effect of ROMO1 on cell viability, flow cytometric analysis was performed to detect cell apoptosis. Compared with the si-NC group, knockdown of ROMO1 significantly promoted apoptosis of HuH-6 cells (*P* < 0.05, Figure 3(d)). In brief, knockdown of ROMO1 restrains cell proliferation and induces apoptosis in HB.

### 3.4. Downregulation of ROMO1 Decreases the Cell Migrative and Invasive Ability in HB

**Figure 4:**
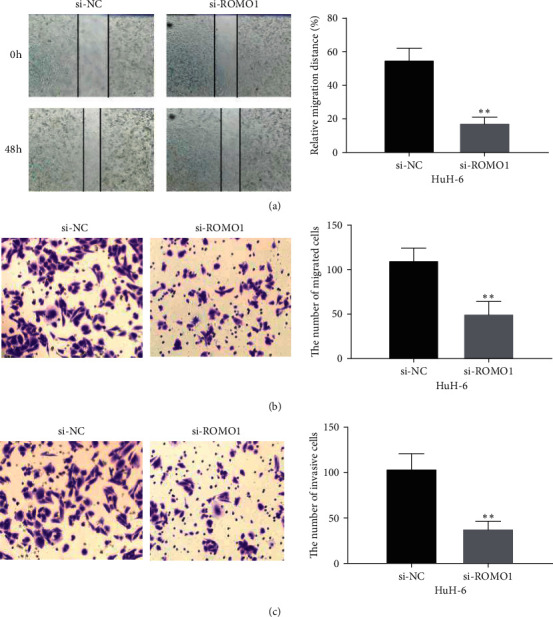
Downregulation of ROMO1 decreases the cell migrative and invasive ability in HB. (a) Wound healing assay showed HuH-6 cell migration capability in the si-ROMO1 or si-NC group. (b, c) Cell migration and invasion were detected in HuH-6 cells with si-ROMO1 or si-NC by the transwell assay. ^*∗∗*^*P* < 0.01.

To investigate how ROMO1 regulates cell migration and invasion, transwell and wound healing assays were performed in HuH-6 cells. We found that the scratch healing ability of HuH-6 cells in the si-ROMO1 group was reduced in comparison with the si-NC group (*P* < 0.05, Figure 4(a)). It indicates that downregulation of ROMO1 can decrease the cell migrative ability of HB cells. In the meantime, transwell assay also showed the same results (*P* < 0.01, Figure 4(b)). In addition, transwell assay also suggested that ROMO1 knockdown significantly restrained cell invasion in HuH-6 cells (*P* < 0.01, Figure 4(c)). All these results revealed that ROMO1 silencing can decrease the cell migrative and invasive ability in HB.

### 3.5. Downregulation of ROMO1 Suppresses Tumor Growth of HB *In Vivo*

**Figure 5:**
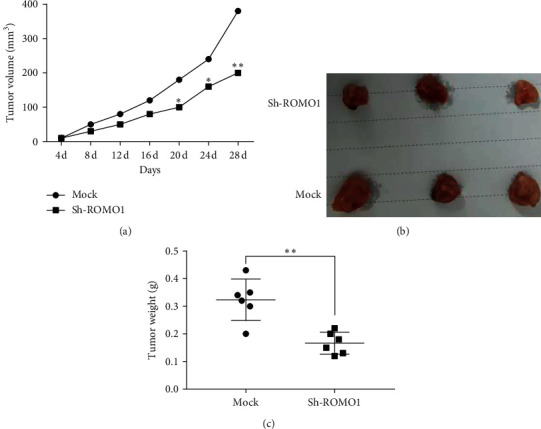
Knockdown of ROMO1 suppresses tumor formation *in vivo*. (a) Tumor size was detected every 4 d. (b) Representative HB tumors at 28 d. (c) Tumor weights were measured in the sh-ROMO1 and Mock group at 28 d. ^*∗*^*P* < 0.05 and ^*∗∗*^*P* < 0.01.

Finally, HuH-6 cells with sh-ROMO1 were injected into nude mice to explore the effect of ROMO1 on tumor growth in HB. We found that tumor growth, as shown by tumor volumes at different time points, was suppressed in the sh-ROMO1 group compared to the Mock group (*P* < 0.05, Figure 5(a)). At the same time, tumor weight in the sh-ROMO1 group was lower than that in the Mock group (*P* < 0.01, Figures 5(b) and 5(c)). The *in vivo* experiment indicates that knockdown of ROMO1 can restrain the tumor formation of HB.

## 4. Discussion

HB accounts for approximately 62% of primary liver malignancies in children. And HB is highly malignant and can be widely metastasized through blood and lymphatic pathways [15]. The first choice of HB treatment is surgical resection, and comprehensive treatment such as corresponding chemotherapy can also be used after the operation [16]. Moreover, the development of HB is slow. When HB is discovered, the tumor is already large. It is difficult to perform operation, and the prognosis of HB is poor [17]. Therefore, the development of new molecular markers for early detection of HB is very important.

Here, we found that ROMO1 was upregulated in LIHC tissues. Upregulation of ROMO1 predicted poor prognosis in LIHC patients. Based on the above results, we speculate that ROMO1 may be involved in the tumorigenesis of HB. As we predicted, upregulation of ROMO1 was also found in HB tissues and cells. Similar to our results, increased expression of ROMO1 was also identified in other cancers, such as bladder cancer [18] and glioblastoma [19]. In addition, it has been reported that high ROMO1 expression predicted unfavorable prognosis and lymphatic metastasis in NSCLC patients [20]. More importantly, ROMO1 has been reported to be a novel potential target for cancer diagnosis and treatment [21]. To investigate whether ROMO1 regulates the tumorigenesis of HB, both *in vitro* and *in vivo* experiments were performed.

Our study demonstrated that ROMO1 knockdown restrained cell proliferation and induced apoptosis in HB. Similar to our results, upregulation of ROMO1 was also found to promote cellular growth in human gliomas [12]. And ROMO1 inhibition induced TRAIL-mediated apoptosis in colorectal cancer [22]. In addition, downregulation of ROMO1 decreased the cell migrative and invasive ability in HB. Lee et al. also reported that ROMO1 and the NF-*κ*B pathway can regulate tumor cell invasion induced by oxidative stress [23]. However, the relationship between ROMO1 and the NF-*κ*B pathway in HB has not been investigated in this study, which needs to be explained in the future. Besides the above results, our study revealed that knockdown of ROMO1 suppressed HB tumor formation *in vivo*. The result has not been found in previous studies.

## 5. Conclusion

Similarly, upregulation of ROMO1 is found in LIHC and predicts poor prognosis in LIHC patients. High expression of ROMO1 is also identified in HB, and it acts as a tumor promoter in the development of HB. Our study provides a potential target for the diagnosis and treatment of HB. However, future investigation is still needed to explain the underlying mechanisms of ROMO1 in HB.

## Data Availability

The datasets used and/or analyzed during the present study are available from the corresponding author upon reasonable request.
